# Chemical Fingerprinting, Aorta Endothelium Relaxation Effect, and Enzymatic Inhibition of Canelo (*Drimys winteri* J. R. Forst. & G. Forst, (D.C) A. Gray, Family Winteraceae) Fruits

**DOI:** 10.3390/foods12132580

**Published:** 2023-07-01

**Authors:** Ruth E. Barrientos, Javier Romero-Parra, Fredi Cifuentes, Javier Palacios, Néstor Jaime Romero-Jola, Adrián Paredes, Gabriel Vargas-Arana, Mario J. Simirgiotis

**Affiliations:** 1 Instituto de Farmacia, Facultad de Ciencias, Universidad Austral de Chile, Valdivia 5090000, Chile; ruth.barrientos@alumnos.uach.cl; 2 Departamento de Química Orgánica y Fisicoquímica, Facultad de Ciencias Químicas y Farmacéuticas, Universidad de Chile, Santiago 6640022, Chile; javier.romero@ciq.uchile.cl; 3 Laboratorio de Fisiología Experimental, Instituto Antofagasta, Universidad de Antofagasta, Antofagasta 1270300, Chile; fredi.cifuentes@uantof.cl; 4 Departamento Biomédico, Facultad Ciencias de la Salud, Universidad de Antofagasta, Antofagasta 1240000, Chile; 5 Laboratorio de Bioquímica Aplicada, Facultad de Ciencias de la Salud, Universidad Arturo Prat, Iquique 1110939, Chile; clpalaci@unap.cl; 6 Departamento de Sanidad Animal, Facultad de Medicina Veterinaria y Zootecnia, Universidad del Tolima, Ibagué 730001, Colombia; njromeroj@ut.edu.co; 7 Laboratorio de Química Biológica, Instituto Antofagasta, Universidad de Antofagasta, Antofagasta 1270300, Chile; adrian.paredes@uantof.cl; 8 Departamento de Química, Facultad de Ciencias Básicas, Universidad de Antofagasta, Antofagasta 1240000, Chile; 9 Laboratorio de Química de Productos Naturales, Instituto de Investigaciones de la Amazonía Peruana, Avenue Abelardo Quiñones, Iquitos 16001, Peru; 10 Facultad de Industrias Alimentarias, Universidad Nacional de la Amazonía Peruana, Iquitos 16001, Peru

**Keywords:** endemic plants, anticholinesterase activity, hypotensive effects, neglected spice, pimiento, Mapuche, Foye

## Abstract

*Drimys winteri* J.R. Forst. & G. Forst (D.C) G. Gray, var. *chilensis* (canelo) is an endemic tree from Chile. Since pre-Columbian times, it has produced a fruit known as the canelo pepper, (pimienta de canelo) or Foye pepper, which can be used as a spice. The chemical and biological analysis of canelo fruits is reported for the first time in this study, that is, its phenolic fingerprinting by UHPLC-PDA- Q-orbitrap MS, the antioxidant activity, the enzymatic inhibitory activity, and its relaxation effects on rat aorta. The proximal composition and the mineral content (Ca: 1.45 ± 0.03 mg/100 g; Mg: 7.72 ± 0.03 mg/100 g; Fe: 4.54 ± 0.21 mg/100 g; Zn: 2.99 ± 0.02 mg/100 g; Mn: 1.08 ± 0.03 mg/100 g; Cu: 0.82 ± 0.02 mg/100 g; K: 53.03 ± 0.20 mg/100 g; Na: 0.087 ± 0.00 mg/100 g) are also reported. The canelo fruits showed a total phenolic content of 57.33 ± 0.82 mg GAE/g dry weight. In addition, the total flavonoid content was 38.42 ± 1.32 mg equivalent of QE/g dry weight. The antioxidant activity was evaluated by employing DPPH and ABTS methods (IC_50_ of 6.65 ± 0.5 and 9.5 ± 0.05 μg/mL, respectively), ORAC (25.33 ± 1.2 μmol Trolox/g dry plant) and FRAP (45.56 ± 1.32 μmol Trolox/g dry plant). The enzymatic inhibition of acetylcholinesterase, butyrylcholinesterase, and tyrosinase (IC_50_: 1.94 ± 0.07, 2.73 ± 0.05, and 9.92 ± 0.05 µg extract/mL, respectively) is also reported. Canelo extract led to an 89% relaxation of rat aorta. Our results confirm that *D. winteri* fruits are a rich source of secondary metabolites and can inhibit enzymes associated with neurodegenerative diseases; the results also suggest that canelo may induce a potentially hypotensive effect in rat aorta. The study demonstrates the medicinal properties of canelo fruit and spice.

## 1. Introduction

**Figure 1:**
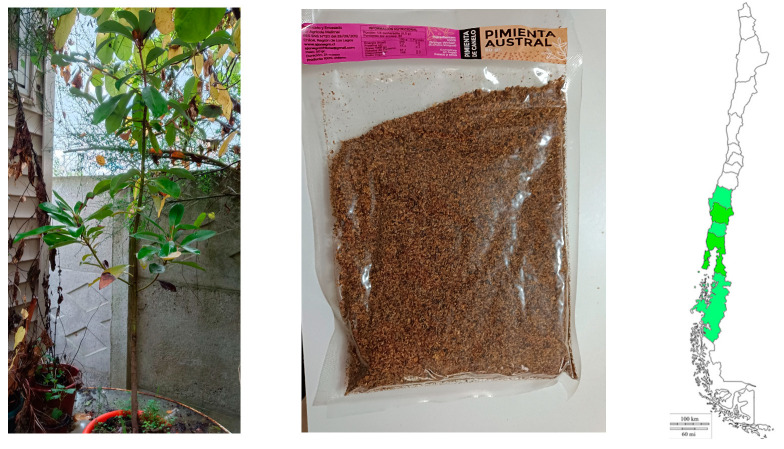
Canelo tree growing in Valdivia, Chile. Sealed commercial product. Zone of distribution of the plant in Chile (green).

Several species of the genus *Drimys*, (Winteraceae) are widely distributed in the Americas (from Mexico in the North to Chile and Argentina in the south). *Drimys confertifolia* Phil. is endemic to the Juan Fernandez Islands, while *Drimys angustifolia* Miers is located in Brasil, *Drimys brasiliensis* Miers is found in Brasil and México, *Drimys andina* (Reiche), R. A. Rodr. and Quez., and syn. *D. winteri* var. *andina* are endemic of Chile, *Drimys granadensis* L.f. is found in México and Perú, and two varieties of *D. winteri* J. R Forst et G. Forst, *D. winteri* var. *winteri*, are found in Southwestern Patagonia (45°44″–55°58′ S). *D. winteri* var. *chilensis* (DC.) A. Gray is widespread across Chile and Argentina (30°2″–46°25″ S). The latter is an evergreen endemic tree that grows in the Coquimbo and Aysén regions of Chile and is most abundant in the Araucania and Los Ríos regions [1,2]. This endemic tree (Figure 1), commonly named canelo in Spanish and Foye in the Mapudungun language, is considered sacred and medicinal in the indigenous Mapuche culture. In traditional Mapuche medicine, a leaf infusion was used to relieve birth and stomach pains, while an infusion of the bark was used to treat cancer and other diseases [3]. Plant species from the genus *Drimys* have been reported to produce bioactive compounds such as essential oils, terpenes, and flavonoids [4]. To date, 18 drimane-type sesquiterpenoids have been characterized for *Drimys winteri* var. *chilensis,* as well as various lignanes such as sesamin, (−) cubebin, and eudesmin. In addition, according to the literature, many flavonoids such as apigenin, luteolin, kaempferol, quercitrin, taxifolin, quercetin, cirsimaritin, fisetin, and astilbin have been reported and isolated but only from bark and leaves of this endemic tree [5].

Our group has previously reported on extracts and some pure compounds from various endemic species in Chile that have potent enzymatic inhibitory effects or other bioactivities linked to ameliorating noncommunicable diseases (NCDs) and neurodegenerative diseases and hypertension. An example is the Chilean *Greigia sphacelata* (Ruiz and Pav.) Regel [6] fruits showed potent inhibition against AChE and BChE, and some metabolites tentatively identified by UHPLC-MS for *G. sphacelata* have been linked to neuroprotective activity. Recently, the berries of *Azara dentata* Ruiz and Pav. [7] were found to have good inhibitory potential against AChE, BChE, and tyrosinase, while some bioactive compounds such as coumarins, anthocyanins, phenolic compounds, and flavonoids, were detected in it by high-resolution UHPLC-MS. The fruits of *D. winteri* are commonly used to produce canelo pepper, which has a slight itch, is a very aromatic spice, and is rarely consumed by humans. The research on canelo has focused on its medicinal character; however, is it necessary to carry out the fingerprinting of chemical constituents and the evaluation of biological activities of canelo fruits with the aim of contributing to the food industry and to the formulation of nutraceuticals or dietary supplements. No previous research about canelo fruit enzymatic inhibition potential or studies relating to its ability to produce vascular relaxation effects have been reported so far.

The goals of this study are to conduct a proximal analysis and to measure the mineral content of *D. winteri* fruits; to analyze the phenolic fingerprint by UHPLC-PDA-MS; to assess canelo fruit antioxidant activity; to evaluate its enzymatic inhibition potential against acetylcholinesterase, butyrylcholinesterase, and tyrosinase; and to determine the hypotensive potential of canelo fruits.

## 2. Materials and Methods

### 2.1. Chemicals

Deionized pure water (<5 µg/L TOC), used for all experiments, was produced in a water system Arium 126 61316-RO coupled to an Arium 611 UV unit (Sartorius, Goettingen, Germany). Methanol (HPLC grade) and formic acid (p.a. for mass spectrometry) were obtained from J. T. Baker (Phillipsburg, NJ, USA. Pure gallic acid (purity > 95%), 2,2′-azinobis(3-ethylbenothiazoline-6-sulfonic acid) diammonium salt (ABTS), 6-hydroxy-2,5,7,8-tetramethylchromane-2-carboxylic acid (Trolox) (purity > 95%), iron (III) chloride hexahydrate, 2,2-diphenyl-1-picrylhydrazyl (DPPH), Folin-Ciocalteu reagent 2,4,6-tri(2-pyridyl)-s-triazine (TPTZ), aluminum chloride, (4-(2-hydroxyethyl)-1-piperazineethanesulfonic acid (HEPES), disodium salt, adenosine 5′-triphosphate (ATP), ethylenediaminetetraacetic acid (EDTA), butyrylcholinesterase from equine serum, and acetylcholinesterase from *Electrophorus electricus* (electric eel) were obtained from Sigma-Aldrich^®^ (Santiago, Chile). Sodium nitrite, sodium carbonate, potassium persulphate, calcium chloride, and sodium hydroxide were obtained from Merck^®^ (Santiago, Chile). Standard grade kojic acid, galantamine, zileuton, sodium acetate trihydrate, plus other HPLC reagents including acetone, chloroform, ethyl acetate, and n-hexane were obtained from Merck^®^ (Santiago, Chile). HPLC standards (quercetin, chlorogenic acid gallic acid, caffeic acid, ferulic acid, vicenin 2, quercetin 3-O-glucoside, isovitexin, orientin, kaempferol, cirsimaritin, artemisinin, vitexin, eriodictyol, purity 95% by HPLC) were obtained from Phytolab (Vesten-Bergsgreuth, Germany), Merck^®^ (Santiago, Chile), Biopurify (Chengdu, China), BOCSY (Shirley, NY, USA), or Extrasynthèse (Genay, France).

### 2.2. Plant Material

Fruits of *Drimys winteri* J.R. Forst. and G. Forst. var. *chilensis* (DC.) A. Gray (family Winteraceae) were collected manually from the months of March–April of the year 2021 in Valdivia, the capital city of the Los Ríos region, near the road on the way to Oncol Park, Chile. The mature fruits were washed, dried at room temperature to obtain pimienta mapuche (Mapuche pepper, Figure 1), and then stored in a dark and cool place sealed without air at an ambient temperature (25 °C). A voucher specimen was placed in the “Laboratorio de Productos Naturales of the Universidad Austral de Chile” (voucher number DW-7-11-21). A total of 5 g of milled canelo fruits were extracted with 20 mL of ethanol: water 1:1 *v*/*v* and the solvent evaporated in vacuo at 45 °C and the remaining water lyophilized to yield 488 mg of a brown extract. The extract was kept at −80 °C in an ultra-freezer for further dissolution and analyses.

### 2.3. Determination of Proximal Composition and Mineral Content

Procedures established by the AOAC were employed in all determinations [8,9]. Moisture content was acquired by drying the fresh fruit samples using an oven at a constant weight. The crude protein content was measured using the Kjeldahl method (N × 6.25) using a Kjelmaster k375 (Büchi Labortechnik AG, Flawil, Switzerland) device and the fiber content by gravimetry after hydrolysis of the samples, while the total lipid content was obtained using the Soxhlet procedure (Sigma-Aldrich, Saint Louis, MO, USA) using petroleum ether as solvent. The ash content was measured using muffle incineration in a furnace at 550 ± 15 °C. Total carbohydrates were 100 − (g water + g protein + g fiber + g fat + g ash). Results are expressed in g per 100 g fresh weight (g/100 g fw). For the mineral content, the spice was dried to ash at 550 °C [9]. The ash was boiled with 20% hydrochloric acid (10 mL) and then filtered and fit to 100 mL with deionized water. Levels of the minerals calcium (Ca), potassium (K), iron (Fe), magnesium (Mg), sodium (Na), zinc (Zn), manganese (Mn), and copper (Cu) were determined using atomic absorption spectroscopy (Varian AA240, Belrose, Australia), previously set with standard solutions with known amounts of the minerals being determined using flames of air-acetylene and nitrous oxide-acetylene, with the latter only being used for calcium analysis. Hollow cathode monometallic lamps were utilized for each element analyzed. All analyses were performed in triplicate.

### 2.4. Ultrahigh Liquid Chromatography Orbitrap MS Analysis (UHPLC OT-MS)

Analytical chromatography was run using a C-18 HPLC column (150 mm × 4.5 mm ID Acclaim, 2.5 µm, Thermo, Bremen, Germany) at 25 °C. The detection wavelengths were 285, 255, 335, and 355 nm, and PDA diode array detectors were set from 200 to 700 nm. Mobile phases were: 2% formic aqueous solution (A) and acetonitrile 2% formic acid (B). The gradient program was first at 5% B (zero time); up to 7% B for 5 min; then to 35% B for 15 min; maintained 35% B for 17 min; went to 80% B for 7 min; maintained 80% B for 10 min; and finally returned to initial conditions in 15 min for column equilibration before the next injection. The flow rate was 0.90 mL/min., and the injection volume was 15 µL. Standards of methanol and the extract were kept at 15 °C in the autosampler. The spectrometer parameters were set as previously reported. Briefly, the flow rate of sheath gas was 75 units; auxiliary gas unit flow rate was 25; capillary temperature was 420 °C; auxiliary gas heater temperature was 520 °C; spray voltage was 2500 V (for ESI^−^); and S lens was RF level 32. Full scan data both positive and negative were acquired at a resolving power of 70,000 FWHM at *m/z* 200, a scan range of *m/z* 70–1000, automatic gain control (AGC) was set at 3 × 10^6^, and the time of injection was set to 200 ms. The system was coupled to MS with a heated ionization HESI II probe. The nitrogen gas (purity > 99.99%) was obtained from a Genius NM32LA (Peak Scientific, Billerica, MA, USA) machine and used as a collision and damping gas. The mass calibration for Orbitrap was performed twice a day to ensure the accuracy of an operating mass equal to 5 ppm. Mass calibration was acquired in both negative and positive modes with an accuracy equal to 5 ppm, as previously reported [6].

### 2.5. Antioxidant Activity and Flavonoid and Phenolics Contents

The total phenolic content (TPC) of *D. winteri* fruit extract was explored by the colorimetric method with Folin–Ciocalteu reagent, and the total flavonoid content (TFC) was acquired by the AlCl_3_ method [10]. The TPC results are expressed as mg gallic acid equivalent (GAE)/100 g dry weight and TFC is expressed as mg quercetin equivalent of QE/100 g dry weight. The measurements were obtained in triplicate and the results reported as the mean ± SD.

The antioxidant capacity of *D. winteri* fruit extract was determined through different methods: DPPH radical inhibition, ABTS radical inhibition, oxygen radical absorbance capacity (ORAC), and Ferric ion reducing antioxidant power (FRAP).

The DPPH inhibition of *D. winteri* fruit extract was obtained according to a method previously reported [11]. The analysis was performed in triplicate and the results reported as IC_50_ (µg extract/mL) of the mean ± SD.

The ABTS inhibition produced by *D. winteri* fruits was measured according to the previous method [12]. The measurements were performed in triplicate. The ABTS results are reported as IC_50_ (µg extract/mL) of the mean ± SD.

The FRAP assay of *D. winteri* was performed as previously described [13]. The analysis was performed in triplicate and the results are expressed as μmol Trolox/g dry plant. The results were acquired by linear regression with Trolox (0–120 μM).

The ORAC capacity was performed according to the previous method described [14]. Values were obtained by a regression equation between the sample concentration or Trolox and area under the fluorescein decay curve. The results are described as μmol Trolox/g dry plant and reported as mean ± SD.

### 2.6. Determination of Inhibitory Enzymatic Activity

Acetylcholinesterase (AChE) and butyrylcholinesterase (BChE) inhibitions were performed in vitro according to the Ellman method. The enzymes were dissolved in Tris-HCl buffer (50 mM, pH 8.0), and 5-dithiol-bis (2-nitrobenzoic acid) was prepared in buffer. The *D. winteri* fruit extract was prepared at a concentration of 2 mg per milliliter in buffer. The sample was mixed with the enzyme. To start the reaction, the respective substrate (acetyl-thiocholine iodide or butyryl-thiocholine chloride) was added. The absorbance at 405 nm was recorded for 30 min at 37 °C. Galantamine was used as the positive control.

The dopachrome method was used for assessing tyrosinase inhibition. In each well were mixed, 20 µL of *D. winteri* fruit extract with 30 µL of PBS (67 mM, pH 6.8), 40 µL of tyrosinase 100 U/mL, and 40 µL of the substrate L-DOPA 2.5 mM. The absorbance was recorded at 492 nm. Kojic acid was the positive control substance.

All measurements of enzymatic inhibition by *D. winteri* fruit extract were acquired in triplicate and the results expressed as IC_50_ (mean ± SD) in µg extract per mL.

### 2.7. Docking Simulations

The partial charges plus the geometries of all compounds shown were fully set using the DFT method with set B3LYP-6-311G+ (d p) as the standard basis [10,15] in Gaussian 09W software [16]. Then, deprotonations and energetic minimizations were acquired using the LigPrep tool in Maestro Schrödinger suite v.11.8 (Schrödinger, LLC, California, USA) [17]. *Torpedo Californica* acetylcholinesterase structure (*Tc*AChE; PDBID: 1DX6 code [18]), plus human butyrylcholinesterase (*h*BChE; PDBID: 4BDS code [19]) and the tyrosinase obtained from *Agaricus bisporus* mushroom (tyrosinase; PDBID: 2Y9X code [20]) were obtained from the Protein Data Bank RCSB PDB [21]. Enzyme optimizations were obtained using the Protein Preparation Wizard from Maestro software, where water molecules and ligands of the crystallographic protein active sites were removed. In the same way, all polar hydrogen atoms at pH 7.4 were added. Appropriate ionization states for acid and basic amino acid residues were considered. The OPLS3e force field was employed to minimize protein energy. The enclosing box size was set to a cube with sides of 26 Å length. The presumed catalytic sites of each enzyme in the centroid of selected residues were chosen, considering their accepted catalytic amino acids: Ser200 for acetylcholinesterase (*Tc*AChE) [22,23], Ser198 for butyrylcholinesterase (*h*BChE) [24,25], and His263 for tyrosinase [20,26,27]. The glide-induced fit docking protocol was employed for the final pairings [28]. Compounds were pointed by the Glide scoring function in the extra-precision mode (Glide XP; Schrödinger, LLC) [29] and were selected by the best scores and best RMS values (cutting criterion: less than 1 unit) to obtain the potential intermolecular interactions between the enzymes and compounds plus the binding mode and docking descriptors. The different complexes were shown using a visual molecular dynamics program (VMD) and Pymol [30].

### 2.8. Isolation of Rat Aorta and Vascular Reactivity Assays

Animals used in this study were male Sprague Dawley rats, aged 6–8 weeks old, *n* = 3, weighing 170–200 g. The experimental procedure was approved (CEIC #366/2022) and performed in accordance with the local ethics research committee of Universidad de Antofagasta. Animals were randomized and caged at room temperature and 45–51% humidity. Rats were maintained with unrestricted access to water and free food. Animals were collected for cervical dislocation. Then, the aortas were kept in a Krebs–Ringer bicarbonate solution, and the aortic rings (2–3 mm) were dissected and placed in an organ bath. Before the assays, the vascular endothelium integrity was assessed with 10^−5^ M acetylcholine (Ach). To evaluate the relaxation effect of the ethanolic extract of *D. winteri* fruits, the aortic rings were precontracted with 10^−6^ M phenylephrine (PE). After the vascular plateau, increasing concentrations of the *D. winteri* extract were added to the organ bath. To evaluate the relaxation role of the endothelium of *D. winteri* extract, the protocol used consisted of removing the endothelium from the aortic rings, then adding increasing concentrations of extract. To evaluate the relaxation effect produced by the fruits, in the organ bath, aortic rings were initially contracted with 10^−6^ M phenylephrine (PE). Once the vascular plateau was reached, cumulative concentrations of *D. winteri* extract were added to the organ bath to record the relaxation effect. The effect of canelo fruit extract on the contractile response to KCl (10–60 mM) and PE (10^−10^–10^−5^ M) was also evaluated using a different methodology. The rings were treated with a single concentration of *D. winteri* fruit extract (100 µg/mL) and incubated for 20 min prior to contraction of the aortic rings with increasing concentrations of KCl or PE.

### 2.9. Statistical Analysis

The data statistical analysis was carried out using 1-way or 2-way analysis of variance (ANOVA) and the post hoc Dunnett test. In addition, the EC_50_ or IC_50_ determination was acquired using sigmoidal nonlinear regression by the Graph Pad Prism version 5.0 software package (GraphPad Software, Inc., La Jolla, CA, USA). Statistical significance was set at *p* = 0.05.

## 3. Results and Discussion

### 3.1. Proximal Composition and Minerals of D. winteri Fruits

Table 1 shows the proximal composition and mineral content of *D. winteri* fruits. It is mandatory to check the full content of metals in foods. The results of a proximal composition showed that canelo fruits are rich in carbohydrates (58%) and have a high protein content (13%), and the mineral content showed that the fruits are rich in magnesium (77 mg/kg) and potassium (530 mg/kg) but lower in sodium, which make this spice very good for the elderly and better than other fruits such as kiwis or local azaras [7]. Black pepper showed 2636.97 mg/kg Ca, 10,006.04 mg/kg K, and 1486.94 mg/kg mg; however, it also showed 1892.12 mg/kg Na [31], while the famous spice Capsicum annuum showed 1160.04 mg/kg Ca, 1253.65 mg/kg mg, and 16,143.58 mg/kg Na [31]. Mg and Ca are important minerals for bone formation, supporting heart functions, relaxing muscle, metabolizing glucose, and conducting memory [32].

### 3.2. HPLC-PDA-MS Identification of the Ethanolic Extract from Canelo Fruits

**Figure 2:**
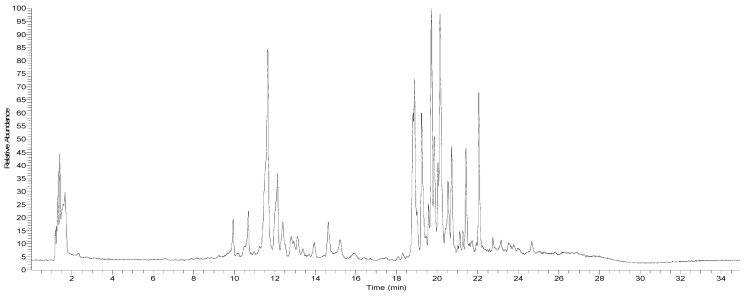
UHPLC Q orbitrap total ion current chromatogram of *D. winteri* dried fruit (pepper) hydroethanolic extract.

**Figure 3:**
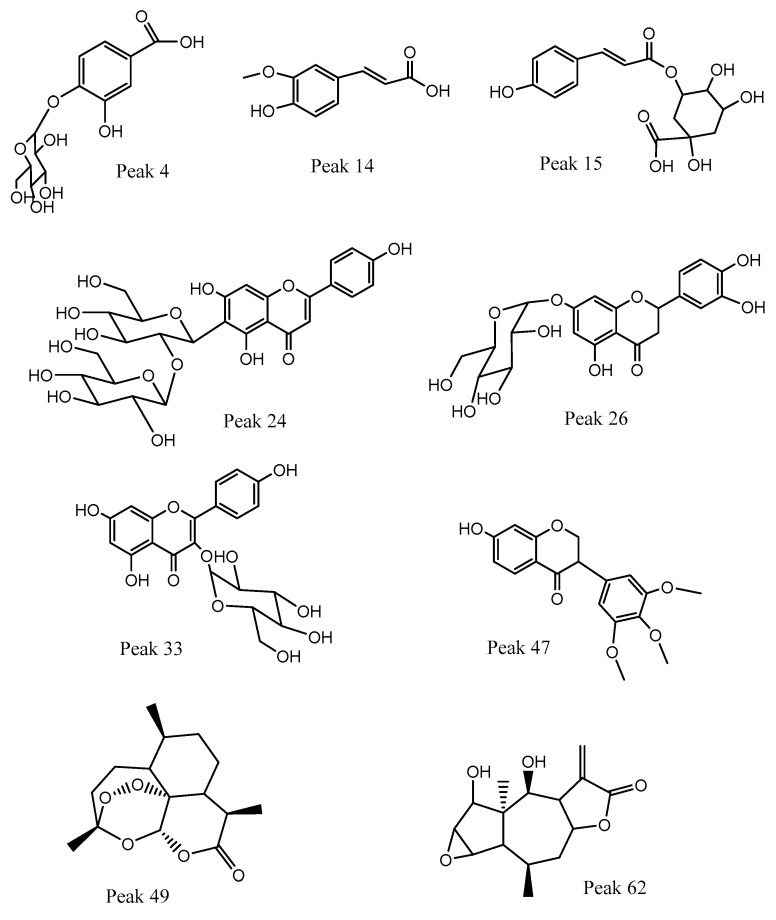
Structures of representative components detected in canelo fruits.

The ethanolic extract of canelo dried fruits (pepper) was analyzed using high-resolution UHPLC Q orbitrap mass analyses. The chromatogram is shown in Figure 2 and the detailed comprehensive MS analysis is shown in Table 2 and commented upon below. Figure 3 shows some structures and Figure S2 (Supplementary Materials) shows full MS spectra of representative compounds.

#### 3.2.1. Phenolic Acids

The presence of several coumaroyl feruloyl or caffeoyl derivatives was detected (Table 2). Caffeoyl quinic acids and their glucosyl derivatives are considered beneficial for human health, mainly due to their antioxidant and anti-inflammatory properties [33]. Peaks 4 and 5 were identified as dihydroxybenzoic acid glucoside (protocatechuic acid 4-O-glucoside, C_13_H_15_O_9_^−^) and hydroxybenzoic acid glucoside (salicylic acid 4-O- glucoside, C_13_H_15_O_8_^−^), while peak 8 was identified as 3,4-dihydroxybenzoic acid (C_7_H_5_O_4_^−^), peak 9 with a pseudomolecular ion at *m/z*: 353.08774 as 3-O-caffeoylquinic acid (C_16_H_17_O_9_^−^), peak 10 as hydroxycaffeoyl quinic acid, peak 11 as caffeoyl acid hexoside (C_15_H_17_O_9_^−^), peak 14 as ferulic acid, peak 15 as 3-O-*p*-coumaroyl quinic acid, peak 16 as quinic acid, peak 17 as 5-O-caffeoyl quinic acid, peak 18 as the dimer derivative caffeoyl quinic acid dimer, peak 19 as p-coumaroyl acid hexoside, while peaks 22, 23, 25, 27, 29, and 30 were caffeic acid, ferulic acid 3-O-glucoside, 5-O-*p*-coumaroyl quinic acid, 3-O-feruloylquinic acid, 4-hydroxycinnamic acid and 5-O-feruloylquinic acid, respectively. Finally, peak 51 was assigned as 2-hydroxyenterodiol and peak 64 as zinniol (C_15_H_21_O_4_^−^).

#### 3.2.2. C-glycosyl Flavonoids

These classes of flavonoids are important in the diet because they showed anti-inflammatory, antidiabetic, anxiolytic, antispasmodic, and hepatoprotective activities [34]. Peak 32 with anion at *m/z*: 431.09727 was assigned as isovitexin (C_21_H_19_O_10_^−^) showing diagnostic C-glycosyl flavone fragments at *m/z*: 341.30762, 323.05405, 311.62509, 283.10669, while peak 36 as its isomer vitexin, and peak 20 as 2″-O-(3″′,4″′-dimethoxybenzoyl) vitexin (C_27_H_31_O_15_^−^). Peak 24 was tentatively identified as isovitexin 2”-O-beta-D-glucoside and peak 44 as isoorientin (luteolin-6-C-glucoside).

#### 3.2.3. O-glycosyl Flavonoids and Aglycones

Several O glycosyl flavonoids were detected, together with some of their aglycones, most of them with a myriad of biological activities [35]. Peak 26 was identified as the flavanone eriodictyol 7-O-hexoside (C_21_H_21_O_11_^−^), peak 28 as myricetin, peak 31 as sophoraflavonoloside (C_27_H_29_O_16_^−^), peak 33 as isoquercitrin and peaks 34, 37, and 43 as kaempferol derivatives: kaempferol-3-O-rutinoside (C_27_H_29_O_15_^−^), kaempferol 3-O-galactopyranoside (C_21_H_19_O_11_^−^), and kaempferol 3-O-pentoside (C_20_H_17_O_10_^−^). Peaks 38–40, 42, 45, 46, 52–55, and 57, 6061, and 65 were assigned as: avicularin, taxifolin, isorhamnetin 3-O-glucoside, luteolin 5-O-glucoside, diosmetin 7-O-beta-D-glucopyranoside, eriodictyol, fisetin, quercetin, isorhamnetin, kaempferol, diosmetin, cirsimaritin, and apigenin 7-O-methyl ether.

#### 3.2.4. Isoflavones

Peak 48 with a parent ion at *m/z*: 329.10651 was identified as 3′-O-methylviolanone (C_18_H_17_O_6_^−^).

#### 3.2.5. Sesquiterpenes

Peak 50 with a pseudomolecular ion at *m/z*: 281.13947 was assigned as the antiparasitic compound artemisinin (C_15_H_21_O_5_^−^) and peak 62 as autumnolide (C_15_H_19_O_5_^−^). Artemisinin is a very active compound and is key for malaria treatment. Its efficacy also broadens to unrelated phylogenetically parasitic infections, such as schistosomiasis [36]. Some drimanes sesquiterpenes isolated from the related species *Drimys brasiliensis* were devoid of antiparasitic activity [37].

#### 3.2.6. Fatty Acids

Peak 57 with an ion at *m/z*: 327.21762, was identified as 9,12,13-trihydroxy-10,15-octadecadienoic acid (C_18_H_31_O_5_^−^), peak 59 as pinellic acid (C_18_H_33_O_5_^−^), and peaks 66 and 67 as octadecanedioic acid (C_18_H_33_O_4_^−^) and hydroxyoctadecatrienoic acid (C_18_H_29_O_3_^−^), respectively.

#### 3.2.7. Other Compounds

Peaks 1–3 were tentatively identified as gluconic malic and citric acids, respectively. Peak 12 was assigned as the aldehyde 3,4-dihydroxybenzaldehyde (C_7_H_5_O_3_^−^). Peak 58 with an ion at *m/z*: 265.14441 was assigned as the β-triketone leptospermone (C_15_H_21_O_4_^−^). Peak 21 was tentatively identified as the lignin eudesmin (C_22_H_25_O_6_^−^). Peak 47 with an ion at *m/z*: 405.17676 was identified as the sugar derivative hexenyl-3-hydroxy-3-methyl-glutaryl hexoside (C_18_H_29_O_10_^−^). Peak 49 with an ion at *m/z*: 447.17923 was assigned as the coumarin lonchocarpenin (C_27_H_27_O_6_^−^).

### 3.3. Total Phenolic and Flavonoid Contents and Antioxidant Activity

Phenolic compounds are one of the biggest groups of natural compounds and in this work the total flavonoid-phenolic contents of *D. winteri* fruits were measured (Table 3). The antioxidant activity of *D. winteri* fruits was determined through different methods (Table 3). The antioxidant capacity of *D. winteri* fruits hydroethanolic extract was good in the different assays evaluated, and this result is supported by the diversity in phenolic and flavonoids detected, as shown in Table 3. Caffeoyl and feruloyl or coumaroyl quinic acid derivatives or glycosides are the main antioxidant compounds [38], together with flavonoids present in fruits and vegetables, and in this study several compounds detected belong to these groups, (TPC 57.33 mg GAE/g and TFC: 38.42 mg QE/g) which support the antioxidant activity of the fruits (Table 3). In comparison, the pericarp of black pepper (Piper nigrum) was reported to contain total phenol-flavonoid contents of 1421.95  ±  22.35 mg GAE/100 g and 983.82  ±  8.19 mg CE/100 g, respectively; however, for normal black and white pepper the TFCs were 11- 654 for black and 447 mg GAE/100 g for white pepper [32].

### 3.4. Enzymatic Inhibitory Activity

Neurodegenerative diseases are increasing worldwide, and the main problem is the disability associated with the disease progression. Common treatments include the use of enzymatic inhibitors agents in Alzheimer’s disease as well as Parkinson’s disease. Cholinergic neurotransmission decreases with the progression of Alzheimer’s disease, and currently, pharmacological treatment (donepezil, galantamine, and rivastigmine) is related to the inhibition of acetylcholinesterase to decrease choline metabolism in the central nervous system, which has the effect of increasing the presence of this neurotransmitter. *D. winteri* fruit extract showed promising results related to the enzymatic inhibitory activity of both enzymes AChE and BChE (Table 4). Moreover, the extract of *D. winteri* causes a potent inhibition of tyrosinase. Previously, our group reported the inhibition of AChE, BChE, and tyrosinase for several endemic and native species from Chile. For example, endemic A. dentata’s berry ethanolic extract showed an IC_50_ of 2.87 ± 0.23 µg/mL, 6.73 ± 0.07 µg/mL, and 9.25 ± 0.15 µg/mL against AChE, BChE, and tyrosinase, respectively [7], while the n-hexane extract of the endemic G. pinifolium plant showed an IC_50_ of 4.58 ± 0.04 µg/mL, 23.44 ± 0.03 µg/mL, and 9.25 ± 0.15 µg/mL against AChE, BChE, and tyrosinase, respectively. G. pinifolium ethyl acetate extract showed AChE (IC_50_ 6.43 ± 0.03 µg/mL), BChE (IC_50_ 33.25 ± 0.02 µg/mL), and tyrosinase (IC_50_ 12.32 ± 0.21 µg/mL) [39]. Canelo fruits showed better activity against AChE and BChE than the bark infusion of native Weinmannia trichosperma, with an IC_50_ of 3.13 ± 0.03 µg/mL for AChE and 2.94 ± 0.08 µg/mL for BChE [40]. In addition, some constituents of canelo detected in this study, such as several caffeoyl quinic acid derivatives, proved previously to have anti AChE and antityrosinase activity [41], as well as some of the flavonoids detected in our canelo fruits such as the flavanone glycoside astilbin [40].

### 3.5. Docking Simulations

**Figure 4:**
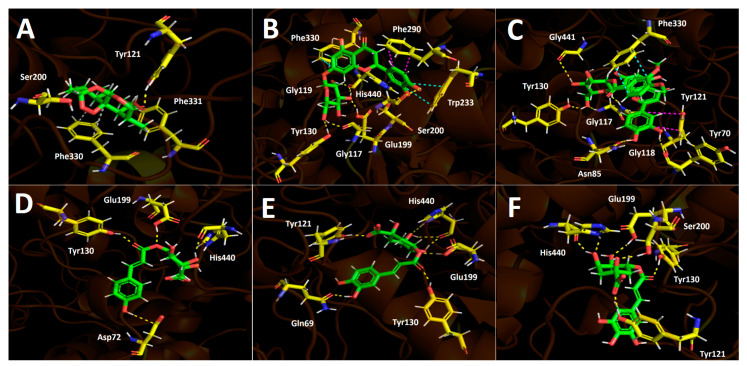
Predicted binding mode and predicted intermolecular interactions between selected compounds in *D. winteri* fruit extract and the residues of *Torpedo Californica* acetylcholinesterase (*Tc*AChE) catalytic site. Yellow dotted lines indicate hydrogen bond interactions, cyan dotted lines represent π-π interactions, magenta dotted lines represent T-shaped interactions, and grey dotted lines represent hydrophobic interactions. (**A**) Artemisinin into the catalytic site; (**B**) eriodictyol 7-O-hexoside into the catalytic site; (**C**) 2″′-O-(3″′,4″′-dimethoxybenzoyl) vitexin into the catalytic site; (**D**) 5-O-p-coumaroyl quinic acid into the catalytic site; (**E**) 3-O-caffeoylquinic acid into the catalytic site; (**F**) hydroxy caffeoylquinic acid into the catalytic site.

**Figure 5:**
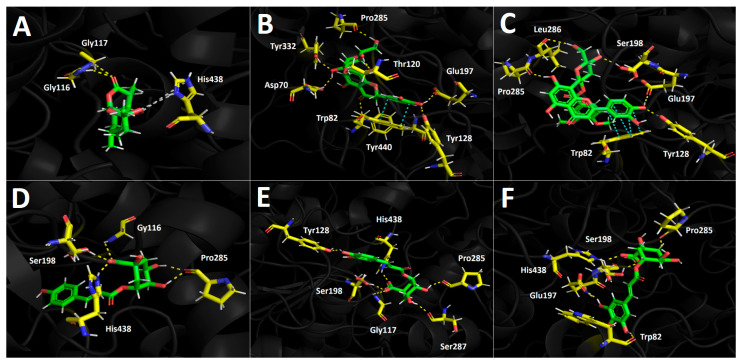
Predicted binding mode and predicted intermolecular interactions between selected compounds in *D. winteri* fruit extract and the residues of human butyrylcholinesterase (*h*BChE) catalytic site. Yellow dotted lines indicate hydrogen bond interactions, cyan dotted lines represent π-π interactions, and grey dotted lines represent hydrophobic interactions. (**A**) Artemisinin into the catalytic site; (**B**) eriodictyol 7-O-hexoside into the catalytic site; (**C**) 2″-O-(3″′,4″′-dimethoxybenzoyl) vitexin into the catalytic site; (**D**) 5-O-p-coumaroyl quinic acid into the catalytic site; (**E**) 3-O-caffeoylquinic acid into the catalytic site; (**F**) hydroxy caffeoylquinic acid into the catalytic site.

Docking simulations were carried out for compounds shown in Figure 4 and Figure 5; each one of them turned out to be the most characteristic and abundant species according to the UHPLC chromatogram (Figure 2) obtained from the fruit extract. Of all compounds identified from the *D. winteri* fruit hydroethanolic extract, artemisinin, eriodictyol 7-O-hexoside, 2″-O-(3″′,4″′-Dimethoxybenzoyl) vitexin, 5-O-p-coumaroyl quinic acid, 3-O-caffeoylquinic acid, and hydroxy-caffeoylquinic acid, as well as the known cholinesterase and tyrosinase inhibitors, galantamine and kojic acid, respectively, were subjected to docking assays into the acetylcholinesterase catalytic site, butyrylcholinesterase catalytic site, and tyrosinase catalytic site. We aimed to analyze the molecular interactions between the different enzymes and the chosen derivatives, as well as to obtain the energy docking descriptors. The latter was performed to rationalize the inhibition activities obtained by the extract (Table 4).

#### 3.5.1. Acetylcholinesterase (TcAChE) Docking Results

All chosen compounds shown in Table 5 mainly perform hydrogen bond interactions with the different residues of the acetylcholinesterase catalytic site. Eriodictyol 7-O-hexoside and 2″-O-(3″′,4″′-dimethoxybenzoyl) vitexin showed the best binding energy values of −18.206 kcal/mol and −16.406 kcal/mol, respectively. On the other hand, the other remaining compounds (artemisinin, 5-O-p-coumaroyl quinic acid, 3-O-caffeoylquinic acid, and hydroxy caffeoylquinic acid) showed moderate binding energies in a range between −8.030 kcal/mol and −12.964 kcal/mol. As was mentioned above, all compounds execute hydrogen bond interactions; nonetheless, eriodictyol 7-O-hexoside and 2″-O-(3″′,4″′-dimethoxybenzoyl) vitexin perform extra π-π and T-shaped interactions, which could explain their energy profiles compared to the other derivatives.

Eriodictyol 7-O-hexoside carries out seven hydrogen bond interactions through its different hydroxyl groups (–OH). Three hydroxyl functions of the saccharide moiety were responsible for five hydrogen bonding interactions, where the involved amino acids were Tyr116, Tyr130, Glu199, and His440. This feature indicates that the polar sugar framework plays an important role in binding energy and acetylcholinesterase inhibition. The other two mentioned hydrogen bond interactions are performed through the two hydroxyl groups that the catechol cores bear and the residues of Gly119 and Ser200 (Figure 4A). Similar to the eriodictyol derivative, the 7-O-hexoside derivative 2”-O-(3”’,4”’-dimethoxybenzoyl) vitexin also executes five hydrogen bond interactions through its hydroxyl functions and the oxygen atom adjacent to the anomeric carbon of the saccharide motif with amino acids Gly117, Gly118, Tyr130, and Gly441, as well as two more interactions with the –OH functions of the catechol at position 3- of the chromone framework (Figure 4B). As previously mentioned, these two compounds perform extra π-π interactions that the others derivatives do not exhibit; in this sense, eriodictyol 7-O-hexoside performs two π-π interactions with Phe330 and Trp233, as well as a T-shaped interaction with Phe330, while 2″-O-(3″′,4″′-dimethoxybenzoyl) vitexin only performs one π-π interaction with Phe330 and a T-shaped interaction with Tyr121, explaining the possible different binding energies observed for both compounds (Table 5).

The structural quinic acid related derivatives 5-O-p-coumaroyl quinic acid, 3-O-caffeoylquinic acid, and hydroxy caffeoylquinic acid displayed similar binding energy values (−10.369 kcal/mol, −12.566 kcal/mol and −12.964 kcal/mol, respectively, see Table 5). 5-O-*p*-coumaroyl quinic acid had the lowest ability to fit into the acetylcholinesterase catalytic site. This could be due to the lower number of hydrogen bond interactions in these derivative forms compared to 3-O-caffeoylquinic acid and hydroxy caffeoylquinic, which exhibit seven and six hydrogen bond interactions, respectively, with acetylcholinesterase catalytic residues. The hydrogen bond interactions carried out by 5-O-*p*-coumaroyl quinic acid are among its two –OH groups (one at the aromatic phenyl ring and the other at the sugar moiety) and the amino acids Asp72 and His440, but two more interactions can be seen between the two oxygen atoms of the ester function and the residues of Glu199 and Ty130 (Figure 4D). The quinic acid-related derivatives, 3-O-caffeoylquinic acid and hydroxy caffeoylquinic acid, are arranged in a similar manner as the acetylcholinesterase catalytic site, nearly overlapping. Therefore, both derivatives perform hydrogen bond interactions with almost the same amino acids: Gln69, Tyr121, Tyr130, Glu199, and His440. It is noteworthy that both derivatives perform hydrogen bond interactions with the residue of Tyr121 through their carboxylate groups presented in their corresponding sugar cores (Figure 4E,F). Finally, artemisinin, due to its hydrophobic structure lacking aromatic rings, shows mainly these types of interactions, which are weaker than hydrogen bond interactions. Thus, artemisinin, exhibiting a −8.030 kcal/mol binding energy, only shows one hydrogen bond interaction with Tyr121 and some hydrophobic interactions with Phe330 and Phe331, among other amino acids.

#### 3.5.2. Butyrylcholinesterase (hBChE) Docking Results

Selected compounds from the *D. winteri* fruit extract subjected to docking experiments on the butyrylcholinesterase catalytic site showed good binding energies. Indeed, Table 4 shows that a canelo half-maximal inhibitory concentration value (BChE IC_50_) is in the same magnitude order as the known inhibitor galantamine (2.73 ± 0.05 µg/mL and 3.82 ± 0.02 µg/mL, respectively). As in acetylcholinesterase docking assays, hydrogen bond interactions predominate among all compounds and the different amino acids of the catalytic pocket. Once again, eriodictyol 7-O-hexoside and 2″-O-(3″′,4″′-dimethoxybenzoyl) vitexin exhibited the best binding energy profiles. Indeed, the structures of these two analogues are hosts to the butyrylcholinesterase catalytic site in similar forms. 5-O-*p*-coumaroyl quinic acid, 3-O-caffeoylquinic acid, and hydroxy caffeoylquinic acid showed similar binding energies, while artemisinin displayed the lowest value (Table 4).

The component 2″-O-(3″′,4″′-dimethoxybenzoyl) vitexin, which was the best compound assayed in our docking experiments in terms of energy, performed five hydrogen bond interactions and one π-π intermolecular bonding. The hydrogen bond interactions of this derivative were executed through the different hydroxyl groups that the chromone, phenyl, and saccharide moieties bear. The implicated residues are Tyr128, Glu197, Ser198, Leu286, and Pro285. Likewise, the observed π-π interaction occurs between the catechol ring and the indole framework of the Tpr82 amino acid (Figure 5C). Considering the similar arrangement between 2″-O-(3″′,4″′-dimethoxybenzoyl) vitexin and eriodictyol 7-O-hexoside in the butyrylcholinesterase catalytic pocket, it can be seen in Figure 5B,C that both derivatives perform their different interactions with many of the same amino acids. In this sense, eriodictyol 7-O-hexoside performs seven hydrogen bond interactions, and six of them are carried out among the hydroxyl groups and amino acids Asp70, Thr120, Tyr128, Glu197, Pro285, and Tyr332. The last one is performed by the oxygen atom of the carbonyl group presented in the chromone structure with Tyr440. As with 2″-O-(3″′,4″′-Dimethoxybenzoyl) vitexin, the π-π interaction executed by eriodictyol 7-O-hexoside occurs between Trp82 and the catechol core. Although eriodictyol 7-O-hexoside performs two more hydrogen bond interactions than 2″-O-(3″′,4″′-Dimethoxybenzoyl) vitexin, the latter exhibits best binding energy (−14.486 kcal/mol and −14.778 kcal/mol, respectively). Nonetheless, the energy descriptors for both derivatives are in the same range and do not represent a significant difference (Table 4).

5-O-p-Coumaroyl quinic acid showed five hydrogen bond interactions, and no π-π interactions were observed in docking results. The hydrogen bond interactions were performed by the hydroxyl groups and the carboxylate function at the sugar core. Indeed, through the two oxygen atoms of carboxylates, three hydrogen bond interactions were observed with residues Gly116, Ser198, and His483. The remaining interactions were performed with the amino acid Pro285 and the two hydroxyls of the sugar motif (Figure 5D). 3-O-Caffeoylquinic acid and hydroxy caffeoylquinic acid, as with eriodictyol 7-O-hexoside and 2”-O-(3”’,4”’-dimethoxybenzoyl) vitexin, showed similar binding energy values (see Table 4). Both quinic acid-related derivatives possess similar structures and settle in a similar manner into the butyrylcholinesterase catalytic site, and thereby shared their hydrogen bond interactions with many residues. 3-O-caffeoylquinic acid performed hydrogen bond interactions with Gly117, Tyr128, Ser198, Pro285, Ser287, and His438, while hydroxy caffeoylquinic acid performed the same type of interactions with the residues of Trp82, Glu197, Ser198, Pro285, and His438 (Figure 5E,F).

Once again, artemisinin showed low binding energy, which could be attributed to the scarce interactions it generates within the catalytic site. In Figure 5A, artemisinin performs two hydrogen bond interactions between the oxygen atom of the ester group and the residues of Gly116 and Gly117, plus hydrophobic interactions with His438, Tro82, and Phe329.

#### 3.5.3. Tyrosinase Docking Results

Inhibition assays of *D. winteri* fruit hydroethanolic extract over tyrosinase showed similar potency to the known inhibitor kojic acid at the same magnitude (9.92 ± 0.05 µg/mL and 3.51 ± 0.02 µg/mL, respectively). In this context, binding energies of selected compounds that underwent docking assays resulted in the same range as the energy of kojic acid (Table 4).

**Figure 6:**
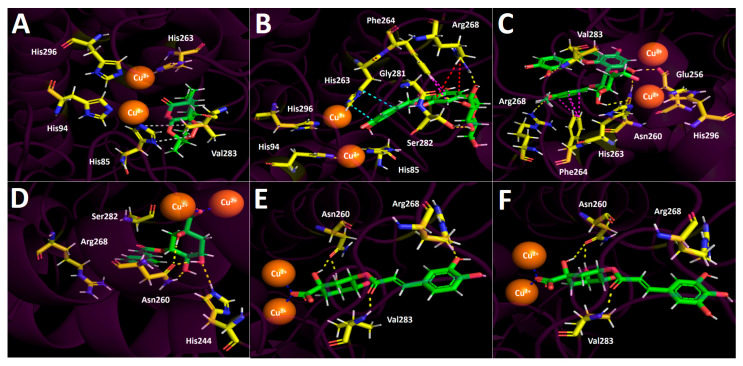
Predicted mode of binding and predicted inter-molecular interactions of selected compounds in *D. winteri* fruit extract and the residues of human *Agaricus bisporus* mushroom tyrosinase catalytic site. Yellow dotted lines indicate hydrogen bond interactions, cyan dotted lines represent π-π interactions, magenta dotted lines represent π-π interactions, red dotted lines represent π-cation interactions, blue dotted lines represent salt bridge interactions, and grey dotted lines represent hydrophobic interactions. (**A**) Artemisinin into the catalytic site; (**B**) eriodictyol 7-O-hexoside into the catalytic site; (**C**) 2″-O-(3″′,4″′-dimethoxybenzoyl) vitexin into the catalytic site; (**D**) 5-O-p-coumaroyl quinic acid into the catalytic site; (**E**) 3-O-caffeoylquinic acid into the catalytic site; (**F**) hydroxy caffeoylquinic acid into the catalytic site.

Concerning the interactions (intermolecularly) of selected compounds, docking descriptors suggest that the principal inhibitory activities would lie in hydroxy caffeoylquinic acid and 5-O-p-coumaroyl quinic acid. On the other hand, artemisinin, as in acetylcholinesterase and butyrylcholinesterase, showed a deficient binding energy of −4.203 kcal/mol, performing mainly hydrophobic interactions such as the one shown with His85 in Figure 6A.

Eriodictyol 7-O-hexoside executes three hydrogen bond interactions with three hydroxyl groups of the saccharide framework and amino acids Ser282, Gly281, and Arg268. Through its aromatic rings (chromone and phenyl scaffolds), this derivative also performs two π-π interactions with His263 and Phe264, as well as a π-cation interaction between the benzene ring of the chromone and Arg268 (Figure 6B). Intermolecular interactions between 2″-O-(3″′,4″′-dimethoxybenzoyl) vitexin and the catalytic residues of tyrosinase are predominantly hydrogen bond interactions, as well as one π-π interaction between the 3,4-dimethoxybenzoyl function (which is attached to one of the oxygens of the sugar moiety) and Phe 264 residue. The amino acids involved in the hydrogen bond interactions mentioned above are Glu256, Asn260, Arg268, and Val283 (Figure 6C).

The quinic acid-related derivatives, 5-O-*p*-coumaroyl quinic acid, 3-O-caffeoylquinic acid, and hydroxy caffeoylquinic acid, are accommodated in the tyrosinase catalytic site in a similar manner. Moreover, since tyrosinase bears two copper cations leading to a binuclear copper binding site, these metal atoms could play a key role in the stabilization of the different tested inhibitors. In the case of the quinic acid-related derivatives, since all of them possess deprotonate carboxylate groups in their structures, this functional chemical group interacts with copper cations in the catalytic site through salt bridge interactions (Figure 6D–F). In addition to the salt bridge interactions of the three derivatives already described all of them also perform hydrogen bond interactions. 5-O-p-coumaroyl quinic acid performs hydrogen bond interactions with His244 and Asn260. Similarly, 3-O-caffeoylquinic acid and hydroxy caffeoylquinic acid perform hydrogen bond interactions with Asn260. Additionally, another hydrogen bond interaction is formed with Val283 through the oxygen atom of the carbonyl group in the aliphatic chain that separates the aromatic ring from the sugar scaffolds of both compounds (Figure 6E,F).

### 3.6. Effect of D. winteri on Vascular Relaxation

**Figure 7:**
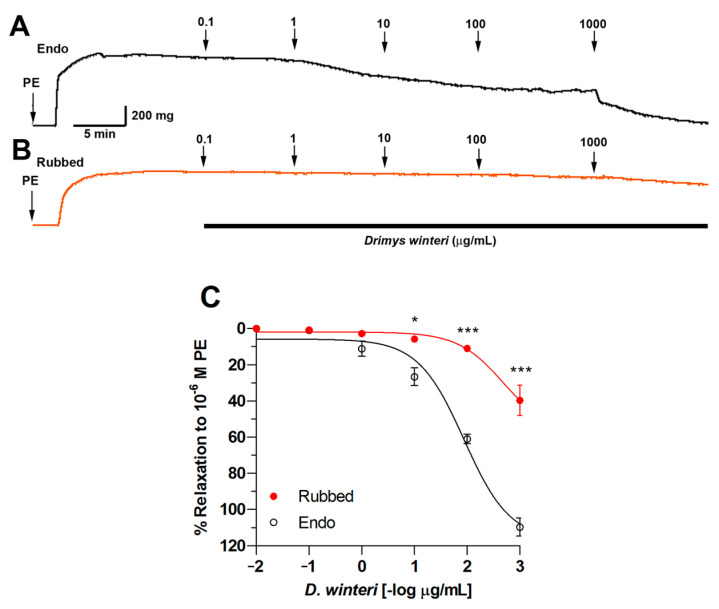
Original record of vascular relaxation using *D. winteri* extract in intact rat aortic rings (Endo) (**A**) or denuded endothelium (Rubbed) (**B**). Concentration-response curve for *D. winteri* fruit ethanolic extract in intact aortic rings (endo) or denuded endothelium (rubbed) (**C**). The rat aorta was pre-contracted with 10^−6^ M PE for 10 min, then every 7 min, rising concentrations of ethanolic extract (0.1 to 1000 µg/mL) were added to the bath. Data are the mean ± SEM of three to five independent experiments. * *p* < 0.05; *** *p* < 0.001 vs. endo.

First, the ability of *D. winteri* fruit to induce vascular relaxation in rat aortic rings was evaluated. Experimental research revealed that *D. winteri* fruit would likely produce a hypotensive effect. The relaxation produced by 100 µg/mL was 64 ± 2%, and when the concentration was increased to 1000 μg/mL, the percentage of vascular relaxation was 115 ± 1%. When the endothelium was denuded of rubbing, vascular relaxation was negligible. The half-maximal effective concentration (EC_50_) for canelo fruit was significantly (*p* < 0.001) different 89 ± 1 µg/mL in intact aorta versus 490 ± 1 µg/mL in denuded endothelium (Rubbed) (Figure 7).

**Figure 8:**
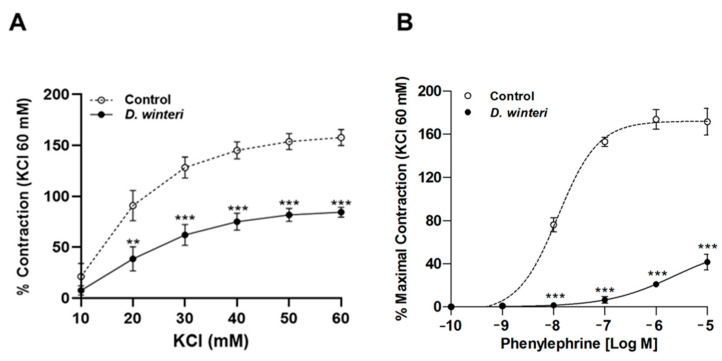
*D. winteri* fruit decreases the vascular contractile response in intact aorta. Tissue was contracted with accumulative concentrations of KCl (10–60 mM) or phenylephrine (from *−*10 to *−*5 [log M]). Aortic rings were pre-incubated with *D. winteri* extract (100 μg/mL) for 20 min, then accumulative concentrations of KCl (**A**) or phenylephrine (**B**) were added. Data are the average ± SEM of four to five independent experiments. ** *p* < 0.01, *** *p* < 0.001 vs. control.

After it was established that *D. winteri* fruit can cause a potential hypotensor effect, further research was conducted to determine whether this effect was linked to vascular contraction. Phenylephrine or potassium chloride were used to cause aortic ring contraction. The maximal vascular contractive response to KCl (60 mM) was decreased by canelo incubation: 158 ± 8% control vs. 84 ± 5% with 100 μg/mL of *D. winteri* ethanolic extract; *p* < 0.001. The half-maximal inhibitory concentration (IC_50_) did not differ between the control and experimental samples (Figure 7). The maximal vascular contractile response to phenylephrine (10^−5^ M) was reduced after incubation with *D. winteri*: 171 ± 12% control vs. 42 ± 7% with 100 μg/mL of *D. winteri* ethanolic extract; *p* < 0.001. In a similar way, the IC_50_ to phenylephrine was significantly different (*p* < 0.05) between control and experimental samples: 176 ± 1 μg/mL control vs. 57 ± 1 μg/mL with 100 μg/mL of canelo fruit extract (Figure 8).

## 4. Conclusions

In this study, the metal content and proximal composition analysis of *D. winteri* fruit are reported for the first time. The chemical fingerprints of canelo fruits, determined by HPLC-MS, are reported. The phenolic fingerprinting yielded 67 compounds, mainly phenolic acids and different types of flavonoids. Interestingly, the antiparasitic compound artemisinin was also detected. This fruit showed interesting contents of phenolics and flavonoids, which is consistent with the number of this class of compounds detected by HPLC-MS. A potent antioxidant activity was found for *D. winteri* fruits, which was evaluated by employing DPPH and ABTS methods combined with ORAC and FRAP. Moreover, the capacity of *D. winteri* fruit extract to induce vascular relaxation in rat aortic rings was evaluated, and the results showed that this food ingredient could produce a potential hypotensive effect because it decreased the vascular response in intact aortic rings in an endothelium dependent manner. The capacity of canelo fruits to inhibit enzymes related to non-communicable diseases, such as Parkinson’s, and Alzheimer’s disease, is reported for the first time. It was observed that *D. winteri* fruit extract inhibits acetylcholinesterase, butyrylcholinesterase, and tyrosinase enzymes. This study highlights the benefits of these native species used as pepper prepared with local Mapuche fruits with nutritional and health-promoting properties and could boost their consumption.

## Figures and Tables

**Table 1:** Proximal composition (%) and mineral content (mg/100 g) of *D. winteri* fruits.

Composition	*D. winteri*
Moisture	15.2 ± 0.10
Ash	6.42 ± 0.26
Fatty matter	0.42 ± 0.02
Crude protein	12.74 ± 0.01
Crude fiber	7.26 ± 0.17
Carbohydrates	57.96 ± 0.01
Minerals	
Ca	1.45 ± 0.03
Mg	7.72 ± 0.03
Fe	4.54 ± 0.21
Zn	2.99 ± 0.02
Mn	1.08 ± 0.03
Cu	0.82 ± 0.02
K	53.03 ± 0.20
Na	0.087 ± 0.00

Each value represents the mean ± SD of triplicates. Values are statistically different (*p <* 0.05).

**Table 2:** HPLC-PDA-MS identification of hydroethanolic extract from canelo fruits (*Drimys winteri*).

Peak Number	Retention Time	UV Max	Tentative Identification	Molecular Formula [M-H]	Measured Mass (*m/z*)	Theoretical Mass (*m/z*)	Accuracy (ppm)	Ions MS^n^
1	1.38	227–272	Gluconic acid	C_6_H_11_O_7_^−^	195.05058	195.04993	3.351	-
2	1.43	229–274	Malic acid	C_4_H_5_O_5_^−^	133.01355	133.01315	3.008	-
3	1.80	227	Citric acid	C_6_H_7_O_7_^−^	191.01933	191.01863	3.684	-
4	2.75	273	Dihydroxybenzoic acid glucoside (Protocatechuic acid 4-O-glucoside)	C_13_H_15_O_9_^−^	315.07248	315.07106	4.509	153.05591, 109.2866, 108.60805
5	2.85	273	Hydroxybenzoic acid hexoside (Salicylic acid 4-O-glucoside)	C_13_H_15_O_8_^−^	299.07718	299.07614	3.461	239.37044, 179.71397, 137.47914, 136.52159, 119.98631
6	3.21	196–227	Unknown	C_18_H_31_O_13_^−^	455.17709	455.17592	2.584	430.82227, 255.95665, 215.00977, 144.00832
7	3.92	196–204–269	Unknown	C_15_H_21_O_7_^−^	313.12918	313.12818	3.198	-
8	5.07	227–265	3,4-Dihydroxybenzoic acid (protocatechuic acid)	C_7_H_5_O_4_^−^	153.01869	153.18242	2.987	-
9	7.15	227–257–312	3-O-Caffeoylquinic acid *	C_16_H_17_O_9_^−^	353.08774	353.08671	2.916	-
10	7.72	227–257–310	Hydroxycaffeoyl quinic acid	C_16_H_19_O_10_^−^	371.09866	371.09727	3.746	354.11142, 341.99585, 191.15846, 173.71552, 135.04477
11	7.86	227–257–312	Caffeoyl acid hexoside	C_15_H_17_O_9_^−^	341.08786	341.08671	3.377	179.78584, 160.84163, 135.04446
12	8.24	227–280–309	3,4-Dihydroxybenzaldehyde	C_7_H_5_O_3_^−^	137.02373	137.02332	2.969	
13	8.47		Unknown	C_19_H_25_O_13_^−^	461.12979	461.12897	1.787	-
14	8.97	214–280–311	Ferulic acid *	C_10_H_9_O_4_^−^	193.05020	193.04954	3.450	-
15	9.06	214–280–312	3-O-*p*-Coumaroyl quinic acid	C_16_H_17_O_8_^−^	337.09283	337.09179	3.087	191.14720, 172.27388, 162.83879, 119.16192
16	9.29	-	Quinic acid	C_7_H_11_O_6_^−^	191.05563	191.05501	3.240	
17	9.30	227–257–310	5-O-Caffeoyl quinic acid	C_16_H_17_O_9_^−^	353.08783	353.08671	3.175	191.05560, 135.04462, 133.99362
18	9.30	227–257–311	Caffeoyl quinic acid dimer	C_32_H_35_O_18_^−^	707.18042	707.18234	2.721	533.58563, 515.55426, 461.33383, 353.01492, 323.06729, 242.81560, 191.05563
19	9.43	214–280–325	p-Coumaroyl acid hexoside	C_15_H_17_O_8_^−^	325.09293	325.09179	3.482	163.03905, 162.83839, 119.04916
20	9.63	247–315–337	2″-O-(3″′,4″′-Dimethoxybenzoyl) vitexin	C_27_H_31_O_15_^−^	595.16620	595.16575	0.760	475.91458, 412.85297, 292.31461
21	9.66	265	Eudesmin	C_22_H_25_O_6_^−^	385.16354	385.16456	−2.651	-
22	9.81	247–267–324	Caffeic acid *	C_9_H_7_O_4_^−^	179.03442	179.03389	3.008	-
23	9.85	247–267	Ferulic acid 3-O-glucoside	C_16_H_19_O_9_^−^	355.10352	355.10236	3.258	193.81488, 178.68074
24	9.93	248–270-332	Isovitexin 2″-O-beta-D-glucoside	C_27_H_29_O_15_^−^	593.15063	593.15010	0.906	413.05600
25	10.15	214–280-323	5-O-p-Coumaroyl quinic acid	C_16_H_17_O_8_^−^	337.09299	337.09179	3.540	173.67874, 163.36201, 119.62347
26	10.26	280	Eriodictyol 7-O-hexoside	C_21_H_21_O_11_^−^	449.10904	449.10784	2.675	423.92548, 301.42798, 287.62851, 259.05487, 174.98462, 151.24983
27	10.49	250–325	3-O-Feruloylquinic acid	C_17_H_19_O_9_^−^	367.10355	367.10236	3.235	191.03110, 178.99828
28	10.50	250	Myricetin *	C_15_H_9_O_8_^−^	317.03009	317.02919	2.828	315.07285, 288.19870, 178.08804
29	10.70	249–283	4-Hydroxycinnamic acid	C_9_H_7_O_3_^−^	163.03955	163.03897	3.558	-
30	10.71	206–249–300	5-O-Feruloylquinic acid	C_17_H_19_O_9_^−^	367.10376	367.10236	3.817	-
31	10.89	251–304–329	Sophoraflavonoloside	C_27_H_29_O_16_^−^	609.14545	609.14557	0.189	255.02881, 151.06294
32	10.98	249–264–334	Isovitexin *	C_21_H_19_O_10_^−^	431.09727	431.09727	2.941	341.30762, 323.05405, 311.62509, 283.10669,
33	11.12	255–300–351	Isoquercitrin (Quercetin 3-O-glucoside)	C_21_H_19_O_12_^−^	463.08832	463.08710	2.888	300.02777, 151.00316
34	11.32	249–281–322	Kaempferol-3-O-rutinoside	C_27_H_29_O_15_^−^	593.15057	593.15010	0.805	287.44540
35	11.33	249–283–324	Unknown	C_19_H_25_O_10_^−^	413.14551	413.14422	3.109	-
36	11.36	248–267–334	Vitexin *	C_21_H_19_O_10_^−^	431.09836	431.09727	2.517	341.05933, 311.35144, 283.06186
37	11.45	250–285–304	Kaempferol 3-O-galactose	C_21_H_19_O_11_^−^	447.09341	447.09219	2.743	327.28265, 284.03296, 255.02979, 226.90129
38	11.53	255–300–351	Avicularin	C_20_H_17_O_11_^−^	433.07779	433.07654	2.890	385.14127, 300.02771, 301.05417, 302.11899, 271.15466, 255.48294, 227.09221, 151.03943
39	11.55	253–288–311	Taxifolin	C_15_H_11_O_7_^−^	303.05078	303.04993	2.811	284.30374, 125.87235
40	11.62	254–354	Isorhamnetin 3-O-glucoside	C_22_H_21_O_12_^−^	477.10400	477.10275	2.623	357.95270, 315.36295, 313.99323
41	11.63	-	Unknown	C_17_H_29_O_10_^−^	393.17676	393.17552	3.139	
42	11.69	256–348	Luteolin 5-O-glucoside	C_21_H_19_O_11_^−^	447.09351	447.09219	2.948	447.09274, 301.03470, 300.02780, 271.02429, 151.00296
43	11.87	265–365	Kaempferol 3-O-pentoside	C_20_H_17_O_10_^−^	417.08279	417.08162	2.808	284.03210, 255.0317, 227.07626
44	11.91	251–288–332	Isoorientin (Luteolin-6-C-glucoside) *	C_21_H_19_O_11_^−^	447.09335	447.09219	2.607	357.40594, 327.08704, 285.13358
45	12.05	250–281–311	Astilbin *	C_21_H_21_O_11_^−^	449.10934	449.10784	3.355	284.37854, 151.61490
46	12.08	251–281	Diosmetin 7-O-glucose	C_22_H_21_O_11_^−^	461.10898	461.10784	2.473	301.03540, 299.37292
47	12.20	-	Hexenyl-3-hydroxy-3-methyl-glutaryl hexoside	C_18_H_29_O_10_^−^	405.17676	405.17552	3.0464	342.00220, 261.29782, 178.37297, 160.84148, 125.87243, 101.02345
48	12.35	250–281-311	3′-O-Methylviolanone	C_18_H_17_O_6_^−^	329.10651	329.10251	−12.139	314.09686, 299.15054, 285.11633, 162.83897, 161.40355
49	13.05	251–267–280	Lonchocarpenin	C_27_H_27_O_6_^−^	447.17923	447.18021	−2.203	215.00941
50	13.20	251–280–285	Artemisinin *	C_15_H_21_O_5_^−^	281.13947	281.13835	3.966	-
51	13.78	252–274–281	2-Hydroxyenterodiol	C_18_H_21_O_5_^−^	317.14285	317.13890	−12,455	298.55157, 287.13138, 267.64462, 258.56961
52	13.83	283	Eriodictyol *	C_15_H_11_O_6_^−^	287.05597	287.05501	3.326	151.46794, 134.95647
53	14.01	253–274–283	Fisetin	C_15_H_9_O_6_^−^	285.04028	285.03936	3.223	-
54	14.38	251–311	Quercetin *	C_15_H_9_O_7_^−^	301.03549	301.03428	4.029	284.31473, 151.00281
55	14.74	252–288–304	Isorhamnetin *	C_16_H_11_O_7_^−^	315.05096	315.04993	3.286	270.46667, 151.20018, 108.47343, 107.73669
56	16.40	251–277–283	9,12,13-Trihydroxy-10,15-octadecadienoic acid	C_18_H_31_O_5_^−^	327.21762	327.21660	3.134	211.13336, 229.13232
57	17.35	249–288	Kaempferol *	C_15_H_9_O_6_^−^	285.04028	285.03936	3.223	133.25186, 117.21797
58	18.10	245–285–311	Leptospermone	C_15_H_21_O_4_^−^	265.14441	265.14344	3.672	251.12869, 249.92970, 196.82272
59	18.28	206–251–286	Pinellic acid	C_18_H_33_O_5_^−^	329.23334	329.23225	3.301	228.57948, 215.00943, 171.10269
60	19.34	254–288–324	Diosmetin	C_16_H_11_O_6_^−^	299.05621	299.05501	4.009	284.03271
61	19.40	253–279	Cirsimaritin *	C_17_H_13_O_6_^−^	313.07178	313.07066	3.554	297.04083, 296.52487, 283.02441
62	19.93	252–286–311	Autumnolide	C_15_H_19_O_5_^−^	279.12387	279.12455	4.194	-
63	19.96	254–290–311	Unknown	C_14_H_19_O_3_^−^	235.13377	235.13287	3.840	191.14365, 163.11220, 144.00845, 119.04957
64	20.15	253–292–311	Zinniol	C_15_H_21_O_4_^−^	265.14450	265.14344	4.017	266.14767, 158.84607
65	20.51	206–255–271	Apigenin 7-O-methyl ether	C_16_H_11_O_5_^−^	283.06116	283.06010	3.735	268.03784
66	21.39	255–292	Octadecanedioic acid	C_18_H_33_O_4_^−^	313.23865	313.23734	4.187	312.97437, 291.35034, 270.84320, 215.00909
67	22.29	255–292	Hydroxyoctadecatrienoic acid	C_18_H_29_O_3_^−^	293.21228	293.21112	3.953	274.50204, 221.15472, 183.12144, 171.08131

* Identified by co-spiking experiments using authentic standard compounds.

**Table 3:** Total phenolic content (TPC), total flavonoid content (TFC), and antioxidant capacities of *D. winteri* hydroethanolic extract.

Assay	TPC ^a^	TFC ^b^	DPPH ^c^	ABTS ^c^	ORAC ^d^	FRAP ^d^
Canelo pepper	57.33 ± 0.82	38.42 ± 1.32	6.65 ± 0.5	9.5 ± 0.05	25.33 ± 1.2	45.56 ± 1.32
Gallic acid	-	-	14.32 ± 0.5	1.67 ± 0.25	-	-

^a^ TPC expressed as mg gallic acid equivalent GAE/g dry weight; ^b^ TFC expressed as mg equivalent of QE/g dry weight; ^c^ IC_50_ expressed as μg/mL; ^d^ expressed as μmol Trolox/g dry plant.

**Table 4:** Enzymatic inhibitory activity (IC_50_ in µg/mL) of *D. winteri* hydroethanolic extract against acetylcholinesterase, butyrylcholinesterase, and tyrosinase.

Assay	AChE	BChE	Tyrosinase
Canelo	1.94 ± 0.07	2.73 ± 0.05	9.92 ± 0.05
Galantamine	0.26 ± 0.02	3.82 ± 0.02	-
Kojic acid	-	-	3.51 ± 0.02

**Table 5:** Binding energies obtained from docking experiments of selected compounds from the *D. winteri* fruit extract, as well as the known inhibitors galantamine and kojic acid over acetylcholinesterase (*Tc*AChE), butyrylcholinesterase (*h*BChE), and tyrosinase.

Compound	Binding Energy (kcal/mol) Acetylcholinesterase	Binding Energy (kcal/mol) Butyrylcholinesterase	Binding Energy (kcal/mol) Tyrosinase
Artemisinin	−8.030	−6.585	−4.203
Eriodictyol 7-O-hexoside	−18.206	−14.486	−8.423
2″-O-(3″′,4″′-Dimethoxybenzoyl) vitexin	−16.406	−14.778	−9.218
5-O-p-Coumaroyl quinic acid	−10.369	−9.509	−10.074
3-O-Caffeoylquinic acid	−12.566	−10.910	−9.898
Hydroxy caffeoylquinic acid	−12.964	−11.425	−10.429
Galantamine	−12.989	−7.125	-
Kojic acid	-	-	−6.050

*Torpedo californica* acetylcholinesterase: *Tc*AChE; human butyrylcholinesterase: *h*BChE.

## Data Availability

Raw HPLC-MS data and other experimental data are available upon request.

## Supplementary Materials

The following supporting information can be downloaded at: https://www.mdpi.com/article/10.3390/foods12132580/s1, Figure S1 (a–h). UHPLC Q Orbitral full MS spectra and structures of representative compounds, Peaks 33, 34, 36, 37, 38, 42, 49 and 50; Figure S2. ACHe inhibition graph by standard Galantamine (IC50 0.266 mg/mL); Figure S3. Trolox curve for ORAC; Figure S4. Gallic acid curve for total phenolic content.
