# Supplementary material for: Chemical Fingerprinting, Aorta Endothelium Relaxation Effect, and Enzymatic Inhibition of Canelo (Drimys winteri J. R. Forst. & G. Forst, (D.C) A. Gray, Family Winteraceae) Fruits
Source: Foods. 2023 Jul 1;12(13):2580. doi: 10.3390/foods12132580 (PMC10340587; doi:10.3390/foods12132580)

Supplementary material for the Article

Chemical Fingerprinting, Aorta Endothelium Relaxation Effect, and Enzymatic Inhibition of Canelo (*Drimys winteri* J. R. Forst. & G. Forst (D.C) Gray, family Winteraceae) Fruits

By Ruth E. Barrientos[1†], Javier Romero-Parra[2†], Fredi Cifuentes[3,4], Javier Palacios[5], Néstor Romero[6], Adrián Paredes[7,8], Gabriel Vargas-Arana[9,10*], Mario J. Simirgiotis[1*]

[1] Instituto de Farmacia, Facultad de Ciencias, Universidad Austral de Chile, Valdivia, 5090000, Chile. ruth.barrientos@alumnos.uach.cl
[2] Departamento de Química Orgánica y Fisicoquímica, Facultad de Ciencias Químicas y Farmacéuticas, Universidad de Chile, Santiago, Chile javier.romero@ciq.uchile.cl
[3] Laboratorio de Fisiología Experimental, Instituto Antofagasta, Universidad de Antofagasta, Antofagasta 1270300, Chile; fredi.cifuentes@uantof.cl
[4] Departamento Biomédico, Facultad Ciencias de la Salud, Universidad de Antofagasta, Antofagasta, 1240000, Chile
[5] Laboratorio de Bioquímica Aplicada, Facultad de Ciencias de la Salud, Universidad Arturo Prat, Iquique, 1110939, Chile clpalaci@unap.cl
[6] Departamento de Sanidad Animal, Facultad de Medicina Veterinaria y Zootecnia, Universidad del Tolima, Ibagué 730001, Colombia njromeroj@ut.edu.co
[7] Laboratorio de Química Biológica, Instituto Antofagasta, Universidad de Antofagasta, Antofagasta 1270300, Chile adrian.paredes@uantof.cl
[8] Departamento de Química, Facultad de Ciencias Básicas, Universidad de Antofagasta, Antofagasta 1240000, Chile
[9] Laboratorio de Química de Productos Naturales, Instituto de Investigaciones de la Amazonía Peruana,
Avenue Abelardo Quiñones, Iquitos 16001, Perú
[10] Facultad de Industrias Alimentarias, Universidad Nacional de la Amazonía Peruana, Iquitos 16001, Perú

Figure S1 (a-h). UHPLC Q Orbitral full MS spectra and structures of representative compounds, Peaks 33, 34, 36, 37, 38, 42, 49 and 50.

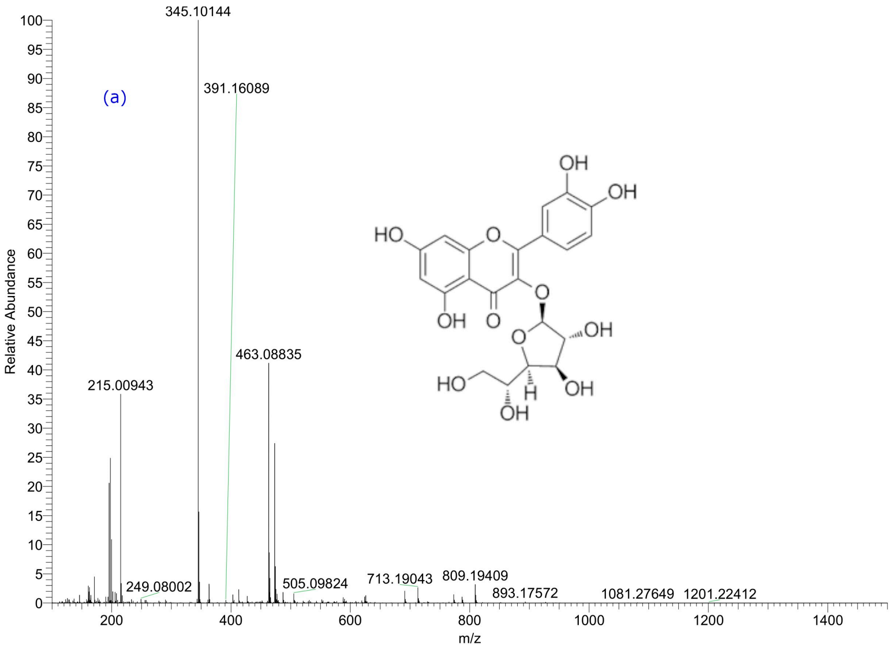

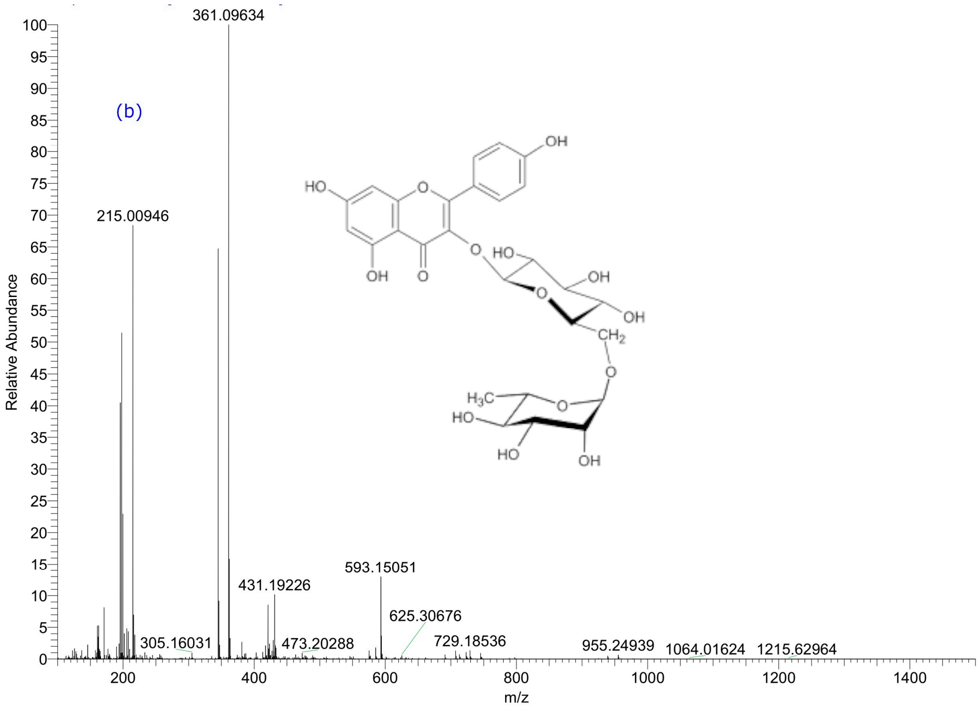

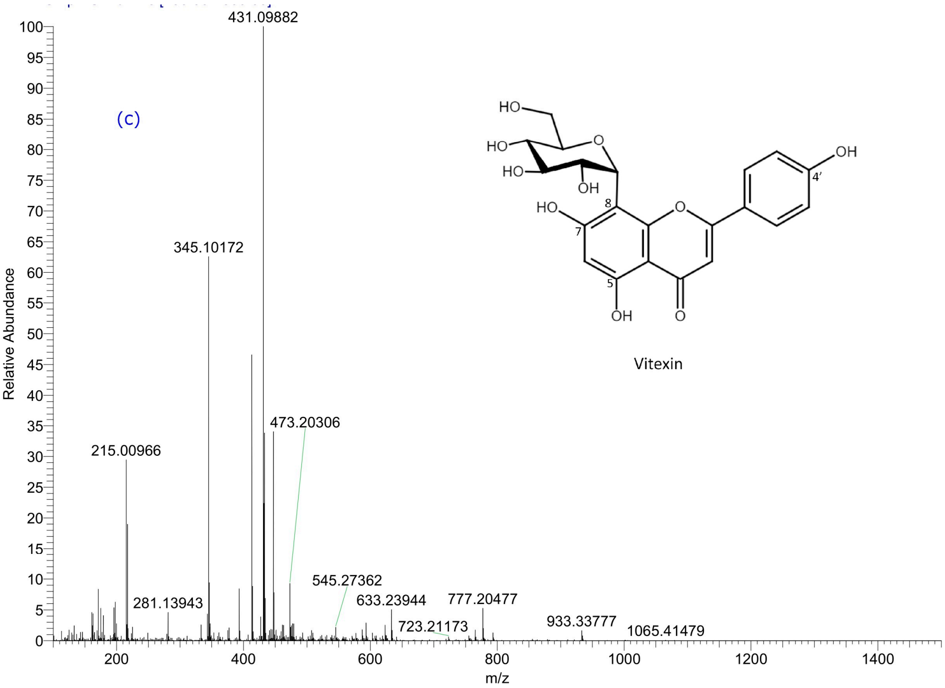

(c)
431.09882
345.10172
473.20306
215.00966
545.27362
281.13943
633.23944
777.20477
723.21173
933.33777
1065.41479
Relative Abundance
m/z
HO
HO
HO
OH
O
OH
HO
8
7
O
5
OH
O
4′
OH
Vitexin

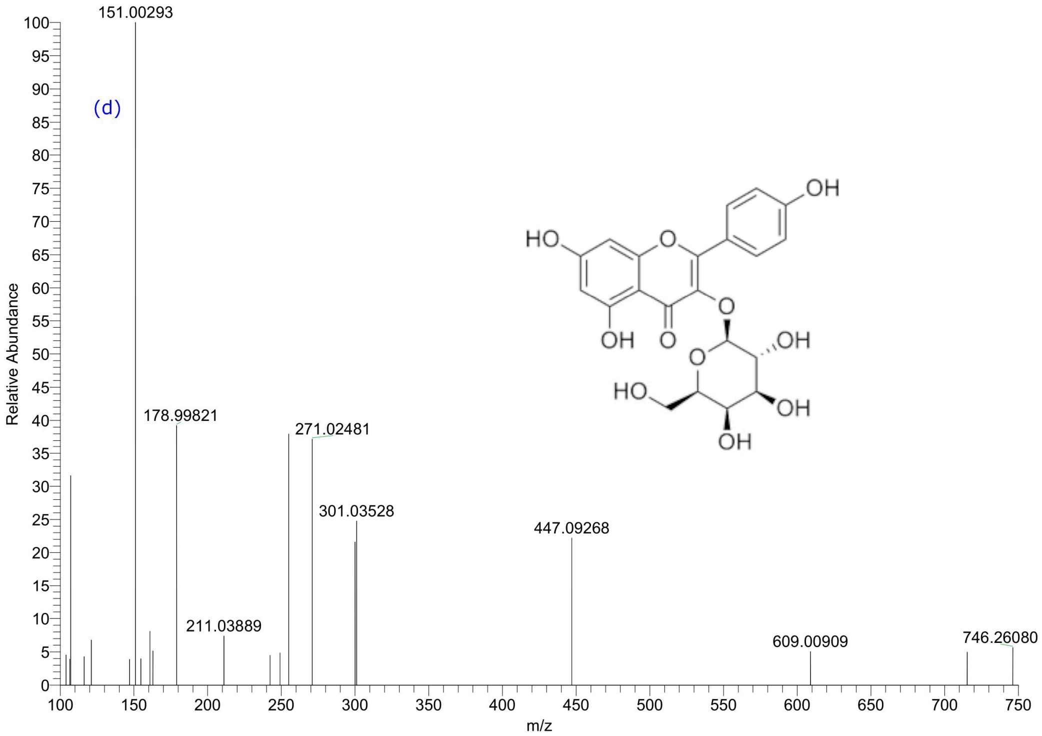

(d)
151.00293
178.99821
271.02481
301.03528
447.09268
211.03889
609.00909
746.26080
Relative Abundance
m/z
HO
O
OH
O
OH
O
O
OH
HO
OH
OH

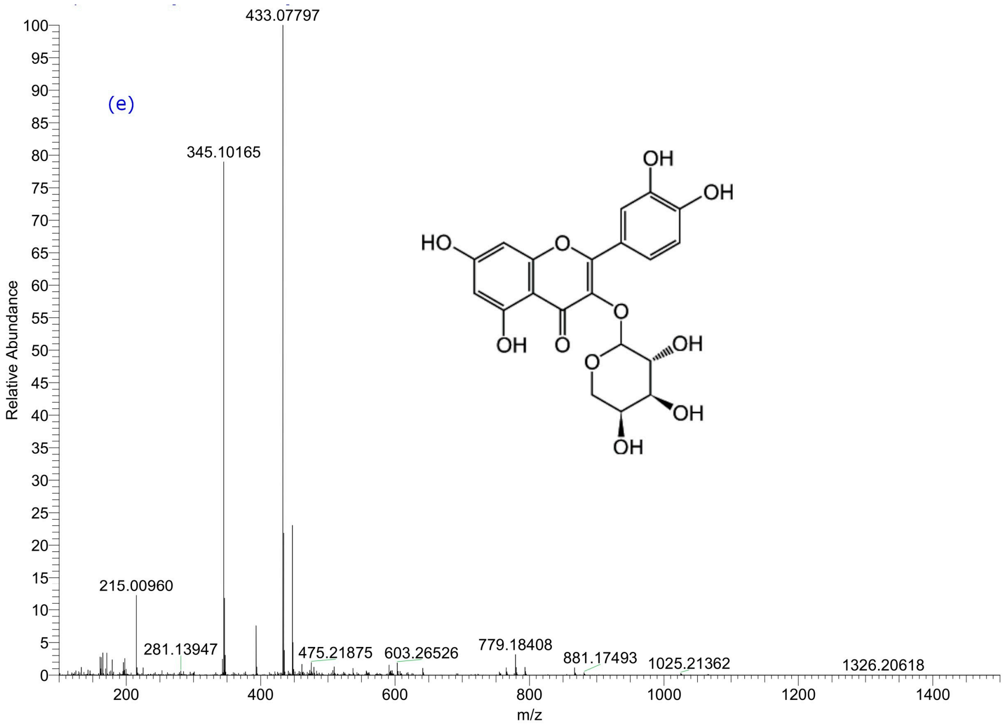
(e)
433.07797
345.10165
215.00960
281.13947
475.21875
603.26526
779.18408
881.17493
1025.21362
1326.20618
Relative Abundance
m/z
HO
OH
O
OH
OH
OH
OH
OH

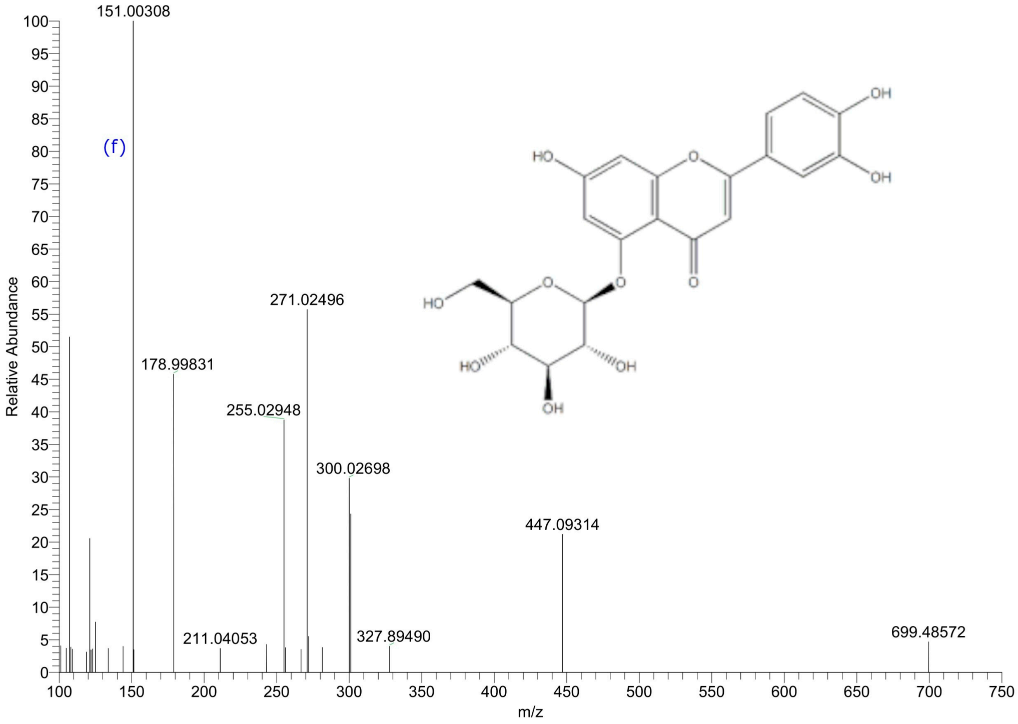
(f)
151.00308
178.99831
271.02496
255.02948
300.02698
447.09314
211.04053
327.89490
699.48572
Relative Abundance
m/z
HO
OH
OH
O
HO
HO
OH
OH

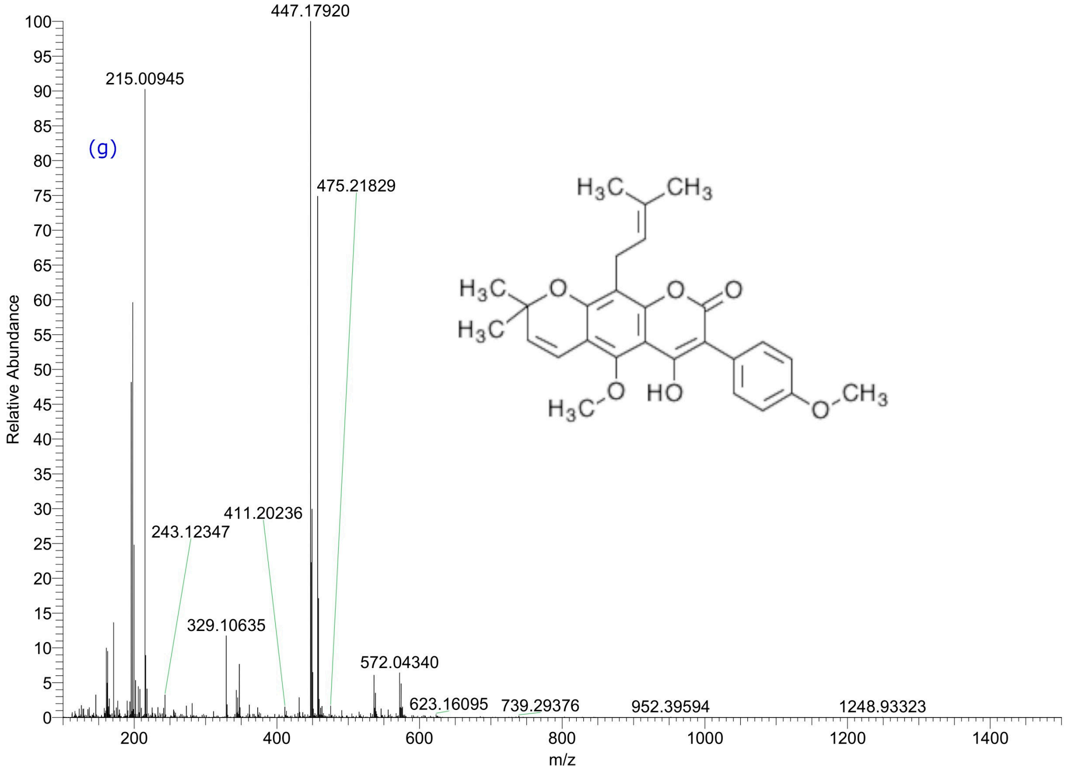
(g)
Relative Abundance
m/z
447.17920
215.00945
475.21829
411.20236
243.12347
329.10635
572.04340
623.16095
739.29376
952.39594
1248.93323
H_3C
CH_3
O
HO
H_3C
O
CH_3

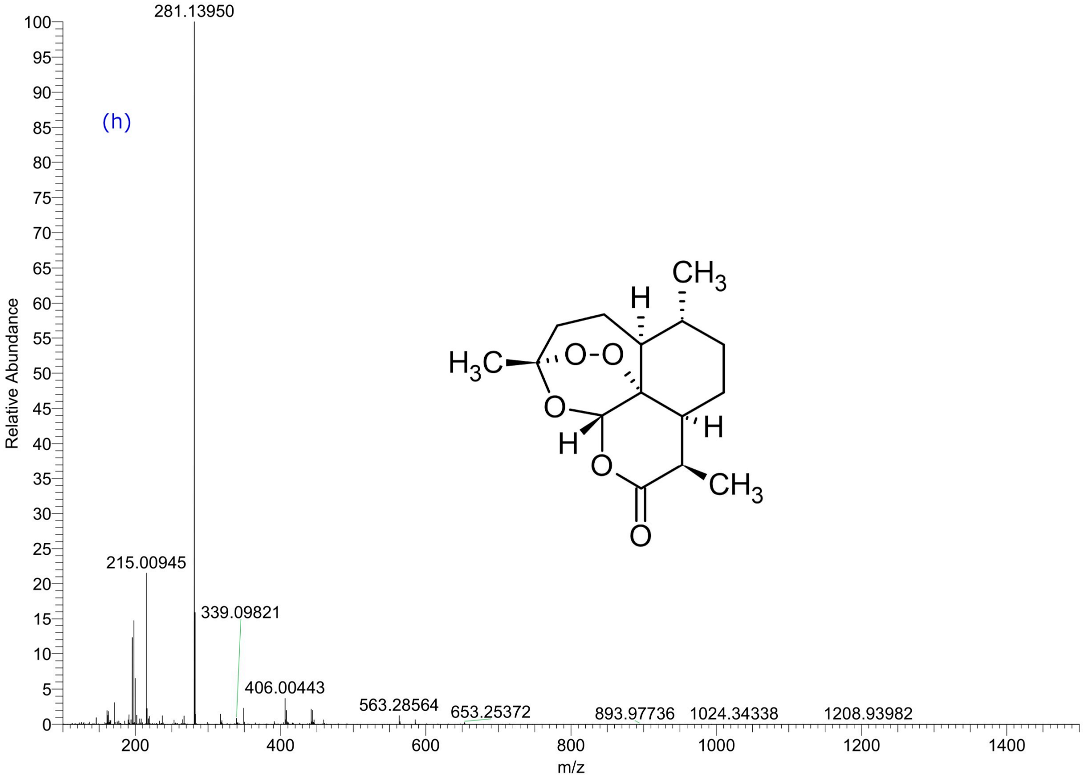
(h)
Relative Abundance
m/z
281.13950
215.00945
339.09821
406.00443
563.28564
653.25372
893.97736
1024.34338
1208.93982
CH_3
H
H_3C
O-O
O
H
O
CH_3
O

Figure S2. ACHe inhibition graph by standard Galantamine (IC50 0.266 mg/mL)

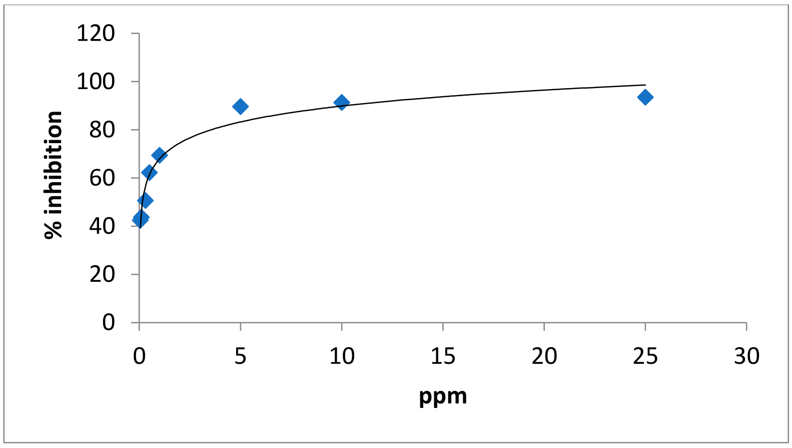

Figure S3. Trolox curve for ORAC

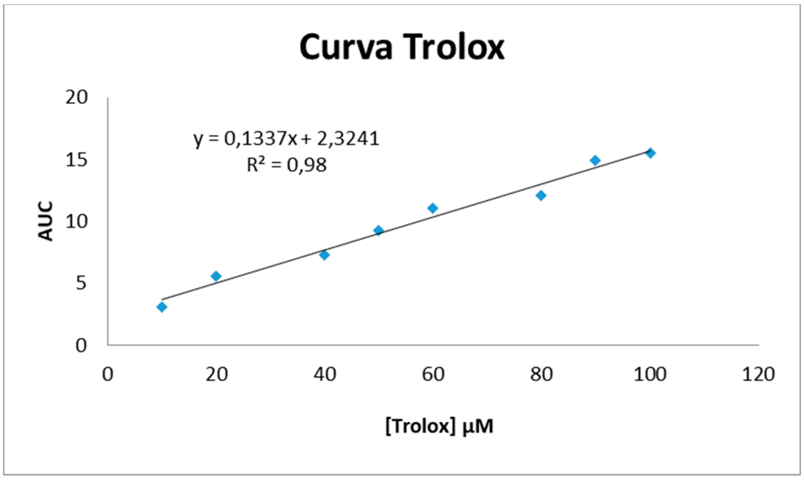

Figure S4. Gallic acid curve for total phenolic content

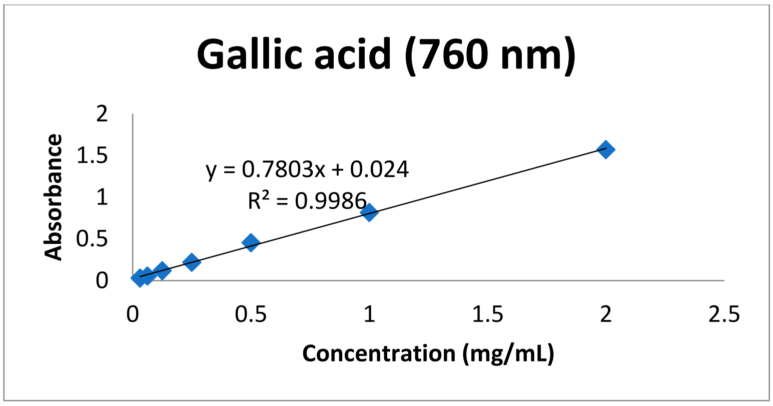

Supplement: Supplementary file 1 [file foods-12-02580-s001.zip › foods-2448311-supplementary.pdf]
